# Immune-checkpoint inhibitor plus chemotherapy versus conventional chemotherapy for first-line treatment in advanced non-small cell lung carcinoma: a systematic review and meta-analysis

**DOI:** 10.1186/s40425-018-0477-9

**Published:** 2018-12-22

**Authors:** Yixin Zhou, Chen Chen, Xuanye Zhang, Sha Fu, Cong Xue, Yuxiang Ma, Wenfeng Fang, Yunpeng Yang, Xue Hou, Yan Huang, Hongyun Zhao, Shaodong Hong, Li Zhang

**Affiliations:** 1 State Key Laboratory of Oncology in South China, Guangzhou, China; 2 Collaborative Innovation Center for Cancer Medicine, Guangzhou, China; 3 Department of VIP region, Sun Yat-sen University Cancer Center, Guangzhou, China; 4 Department of Radiotherapy, Sun Yat-sen University Cancer Center, Guangzhou, China; 5 Department of Medical Oncology, Sun Yat-sen University Cancer Center, 651 Dongfeng East Road, Guangzhou, 510060 China; 6 Guangdong Provincial Key Laboratory of Malignant Tumor Epigenetics and Gene Regulation, Pathology Department, Sun Yat-Sen Memorial Hospital, Sun Yat-Sen University, Guangzhou, China

**Keywords:** Immune checkpoint inhibitor, Programmed death 1, Programmed death 1 ligand 1, Chemotherapy, Non-small cell lung carcinoma, Predict

## Abstract

**Background:**

Immune-checkpoint inhibitors plus chemotherapy are emerging as effective first-line treatment in advanced non-small-cell lung carcinoma (NSCLC), but little is known about the magnitude of benefits and potential clinical predictors.

**Methods:**

We performed a meta-analysis of randomized trials that compared PD-1/PD-L1 inhibitor plus chemotherapy with chemotherapy in first line of treatment for advanced NSCLC. The outcomes included progression-free survival (PFS), overall survival (OS), objective response rate (ORR) and treatment-related adverse events (AEs). A fixed-effect or random-effects model was adopted depending on between-study heterogeneity.

**Results:**

Six trials involving 3144 patients were included. PD-1/PD-L1 inhibitor plus chemotherapy was significantly associated with improvement of PFS (hazards ratio [HR], 0.62; 95% CI 0.57–0.67; *P* < .001), OS (HR, 0.68; 95% CI 0.53–0.87; *P* = .002) and ORR (relative ratio [RR], 1.56; 95% CI 1.29–1.89; *P* < .001), irrespective of PD-L1 expression level. The significant predictor(s) for treatment benefit with combination therapy versus chemotherapy alone were PD-L1 expression level for PFS (*P* < .001); types of checkpoint inhibitor for ORR (*P* < .001); histology (*P* = .025), age (*P* = .038), gender (*P* < .001), and types of checkpoint inhibitor (*P* < .001) for OS. In safety analyses, PD-1/PD-L1 inhibitor plus chemotherapy had significantly higher incidence of adverse events (AEs) of grade 3 or higher (RR, 1.14; *P* = .007), AEs leading to treatment discontinuation (RR, 1.29; *P* = .022), serious AEs (RR 1.70; *P* = .006), immune mediated AEs of any grade (RR, 2.37; *P* < .001), and immune mediated AEs of grade 3 or higher (RR, 3.71; *P* < .001).

**Conclusions:**

PD-1/PD-L1 inhibitor plus chemotherapy, compared with chemotherapy, is associated with significantly improved PFS, ORR, and OS in first-line therapy in NSCLC, at the expense of increased treatment-related AEs.

**Electronic supplementary material:**

The online version of this article (10.1186/s40425-018-0477-9) contains supplementary material, which is available to authorized users.

## Background

Advanced non-small-cell lung cancer (NSCLC) remains the leading cause of cancer-related mortality worldwide [1, 2]. Platinum-based chemotherapy has been the standard of care for the first-line treatment of advanced NSCLC that lacks targetable driver mutations [3]. However, chemotherapy is associated with only modest efficacy and has reached a plateau. With recent advance of immune checkpoint inhibitors treatment that block the PD-L1 (programmed cell death 1 ligand 1) and PD-1 (programmed cell death 1) pathway, pembrolizumab monotherapy has replaced chemotherapy as the first-line treatment for patients with PD-L1 tumor proportion score (TPS) of at least 50% [4], and pembrolizumab plus platinum and pemetrexed for those with nonsquamous histology irrespective of PD-L1 expression [5, 6].

Preclinical evidence suggested that chemotherapeutic agents may exert immune-potentiating effects under certain condition [1], exemplified by increasing the mutational load in cancer cells which leads to a higher chance of neoantigen presentation [7], augmenting major histocompatibility complex class I and human leucocyte antigen (HLA)-A, B, C expression [8, 9], reducing the activity of immune-suppressive cells [10, 11], and increasing the sensitivity of tumor cells to T-cell effector cytokines [12]. Chemotherapy has also been shown to induce PD-L1 expression on tumor cells [13, 14]. Thus, combining immunotherapy and chemotherapy might synergistically improve the antitumor activity of anti-PD-1 and anti-PD-L1 monotherapy, which have been demonstrated in several randomized controlled trials [5, 6, 15].

Despite the promising activity of immuno-oncology (IO) combinatorial treatment, there remain several unanswered questions. For example, will the IO-chemotherapy combinatorial regimens lead to improved efficacy in all comers at the expense of increased toxicity? Are there any clinical or molecular factors that could predict benefit of IO-chemotherapy combination?

Because the magnitude of IO-chemotherapy benefits remains controversial and individual trials were not powerful enough to explore a difference of treatment effect between patient subgroups, a meta-analysis of currently available trials comparing PD-1/PD-L1 inhibitor plus chemotherapy with chemotherapy will provide important and clinically useful information.

## Method

### Study eligibility and identification

Eligible randomized controlled trials that compared PD-1/PD-L1 inhibitor plus chemotherapy with chemotherapy in the first-line setting were identified from Pubmed, Embase and the Cochrane Central Register of Controlled Trials. We searched for studies that published in English from inception to June 10, 2018, using the keywords including pembrolizumab, nivolumab, atezolizumab, durvalumab, PD-1, PD-L1, non–small cell lung cancer, and randomized controlled trial (Additional file 1: Supplementary Method). We also reviewed abstracts from major conference proceedings of the American Society of Clinical Oncology (ASCO), the European Society of Medical Oncology (ESMO), the American Association for Cancer Research (AACR), and the World Conference on Lung Cancer (WCLC). When duplicate studies were identified, only the most complete and updated data of the study was included.

### Data extraction

Two authors (Y.X.Z. and C.C.) independently extracted data with a predefined information sheet. Discrepancies were resolved by consensus. We extracted the following items for each included trial: acronym and design of the trial, number of patients enrolled, year of publication or conference presentation, clinicopathological characteristics of the patients including PD-L1 level, type of chemotherapeutic agents and IO drug, and treatment outcomes including progression-free survival (PFS), overall survival (OS), objective response rate (ORR), duration of response, and treatment-related adverse events (AEs). When we needed additional information that were not provided, we contacted the corresponding authors to request it. Two independent reviewers (S.D.H. and X.Y.Z.) conducted the risk of bias assessment of the included trials with the Cochrane Collaboration’s tool [16].

### Statistical analysis

The primary objective was to investigate the association between IO-chemotherapy vs. chemotherapy and treatment effects (PFS, OS, and ORR) in patients with advanced NSCLC. The PFS and OS outcomes were measured with hazards ratios (HRs) and the corresponding 95% confidence intervals (CIs) which were extracted from each study or calculated using other available statistics. The secondary outcome was the pooled risk of adverse events.

We used the Cochrane’s Q statistic to assess between-study heterogeneity, and calculated the *I*^2^ statistic, which estimates the percentage of total variation across studies due to heterogeneity rather than chance. The pooled estimates for PFS and OS were presented with HRs, 95% CIs and *P* values calculated using the inverse-variance-weighted method, while the measures for dichotomous data (ORR and frequency of adverse events) were pooled with the risk ratios (RRs), 95% CIs and *P* values using the Mantel Haenszel method. The random effect models were chosen if obvious heterogeneity was present (*I*^2^ > 50%), otherwise the fixed effect models were applied [16].

To investigate the sources of heterogeneity, predefined subgroup analyses were performed. Tests of interaction were used to assess the differences in treatment effect across these subgroups. Publication bias was evaluated by examining the funnel plot of the effect size for each trial against the reciprocal of its SE, together with the Egger test. Sensitivity analyses of treatment efficacy were also conducted by: 1) repeating the analyses by omitting one study at a time; 2) repeating the analysis by removing studies that were only available from conference presentation; and 3) using both fixed-effects and random-effects models for the analysis. We used Stata version 15.0 (Stata Corporation, College Station, TX) for all of the analyses. The nominal level of significance was set at 5%.

## Results

A total of 1329 studies were identified through the initial search strategy. After screening the abstracts and reviewing the full texts, a total of six trials [5, 6, 15, 17–20] involving 3144 patients were included in the final analyses (Additional file 2: Figure S1). The assessment of risk of bias was provided in Additional file 3: Table S1. All the trials were well designed and reported. The main source of bias was that data in three trials (CheckMate 227, KEYNOTE-407, and Impower131) could only be retrieved from conference presentations [17–19].

The main characteristics of the included trials were summarized in Table 1 and Additional file 3: Table S2. The patient characteristics were well balanced between the experimental and control groups in all trials. Three trials [5, 6, 15, 20] enrolled patients with nonsquamous NSCLC, two trials [17, 19] with squamous NSCLC, and one trial [18] with both squamous and nonsquamous patients. Four trials [5, 6, 18–20] investigated anti-PD-1 antibody and two trials [15, 17] investigated anti-PD-L1 antibody. All six trials used standard-of-care chemotherapeutic regimens as recommended by practice guidelines.

**Table 1:** Characteristics of Patients Comparing IO-Chemotherapy with Chemotherapy in Included Trials

Source	PD-(L)1 Drug^b^	Histology	No. of patients^a^		Median age (years)^a^	Male (%)^a^	Performance status^a^		PD-L1 subgroups^a^		
			ITT	As treated			ECOG 0 (%)	ECOG 1 (%)	<1% (%)	1–49% (%)	≥50% (%)
KEYNOTE-189 2018 [6]	Pembrolizumab	nonsquamous	410 vs 206	405 vs 202	65 vs 64	62 vs 53	45 vs 39	54 vs 61	31 vs 31	31 vs 28	32 vs 34
IMpower150 2018 [15]	Atezolizumab	nonsquamous	400 vs 400	393 vs 394	63 vs 63	60 vs 60	39 vs 43	60 vs 57	47 vs 50	33 vs 31	20 vs 19
KEYNOTE-021 2016 [5], 2018 [20]	Pembrolizumab	nonsquamous	60 vs 63	59 vs 62	63 vs 63	37 vs 41	40 vs 46	58 vs 54	35 vs 37	32 vs 37	33 vs 27
KEYNOTE-407 2018	Pembrolizumab	squamous	278 vs 281	278 vs 280	65 vs 65	79 vs 84	26 vs 32	74 vs 68	34 vs 35	37 vs 37	26 vs 26
IMpower131 2018 [17]	Atezolizumab	squamous	343 vs 340	334 vs 334	65 vs 65	81 vs 82	34 vs 32	66 vs 68	47 vs 50	38 vs 36	15 vs 14
CheckMate 227 2018 [18]	Nivolumab	suqamous and nonsquamous	177 vs 186	172 vs 185	64 vs 64	73 vs 67	33 vs 31	66 vs 68	100 vs 100	0 vs 0	0 vs 0

^a^Data presented as “IO-chemotherapy group vs chemotherapy group”

^b^Pembrolizumab (200 mg, Q3W), Atezolizumab (1200 mg, Q3W), Nivolumab (360 mg, Q3W)

*Abbreviation*: *IO* immuno-oncology, *ITT* intention-to-treat

The main outcomes of the included trials were summarized in Additional file 3: Table S3. The median follow-up time ranged from 7.8 to 23.9 months. All six trials provided PFS, ORR and DOR data; OS data was not reported in CheckMate 227 study.

### Benefit of IO-chemotherapy combination

The pooled result showed that IO-chemotherapy combination significantly reduced the risk of disease progression compared with chemotherapy (HR, 0.62; 95% CI 0.57–0.67; z = 11.06, *P* < .001) (Additional file 2: Figure S1A). There was no significant heterogeneity in the overall treatment effect in terms of PFS across the six trials (*I*^2^ = 42.3%, χ^2^ = 8.66, *P* = .123). The funnel plot for the PFS revealed no asymmetry (Additional file 2: Figure S2A; Egger test *P* = .713), indicating no obvious publication bias regarding PFS.

**Fig. 1:**
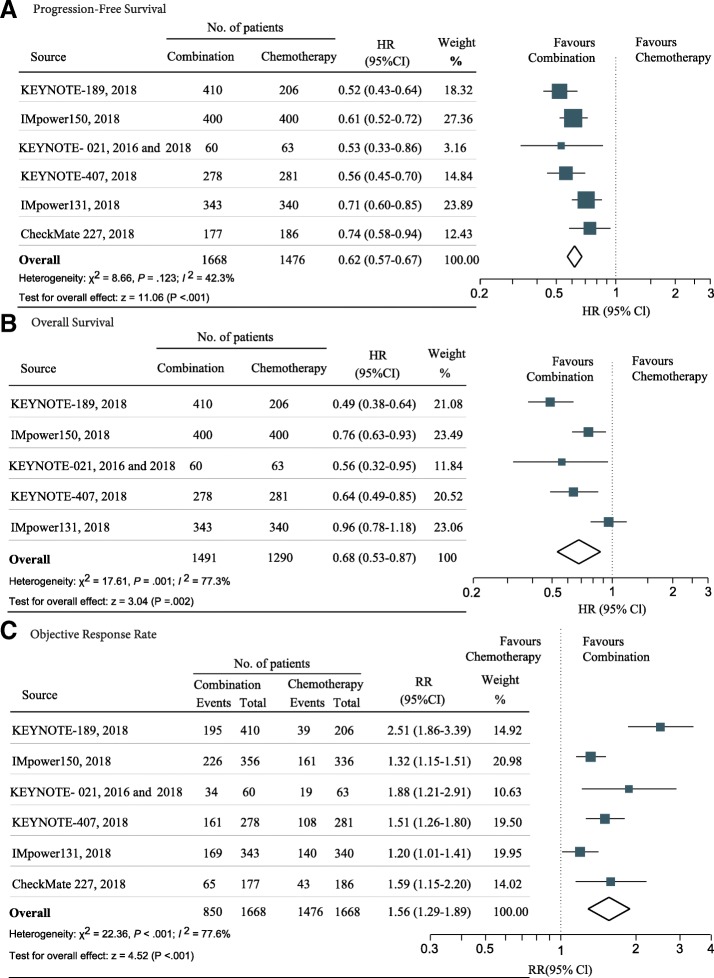
Forest plot of hazard ratios and risk ratios comparing (**a**) progression-free survival, (**b**) overall survival, and (**c**) objective response rate in patients who received IO-Chemotherapy vs Chemotherapy alone. Squares represent study-specific effect size (HR or RR). The area of square is inversely proportional to the standard error of the study (and therefore indirectly to the sample size) and larger area indicates greater weight in the calculation of the pooled effect size. The horizontal line crossing the square represents the 95% CI. The diamonds represent the estimated overall effect, based on the meta-analysis. HR, hazard ratio; RR, relative risk; CI, confidence interval

In terms of OS benefit, the IO-chemotherapy combination led to a 32% reduction in the risk of death compared with chemotherapy (HR, 0.68; 95% CI 0.53–0.87; z = 3.04, *P* = .002) (Fig. 1b). The objective response rate was also significantly improved with the IO-chemotherapy combination (pooled RR, 1.56; 95% CI 1.29 to 1.89; z = 4.52, *P* < .001) (Fig. 1c). Significant heterogeneity was observed in the analysis of OS (*I*^2^ = 77.3%, χ^2^ = 17.61, *P* = .001) and ORR (*I*^2^ = 77.6%, χ^2^ = 22.36, *P* < .001), respectively. The funnel plots for the OS and ORR revealed no asymmetry (Additional file 2: Figures S2B and C; Egger test: *P* = .370 for OS analysis and *P* = .308 for ORR analysis, respectively).

### Subgroup analyses

**Fig. 2:**
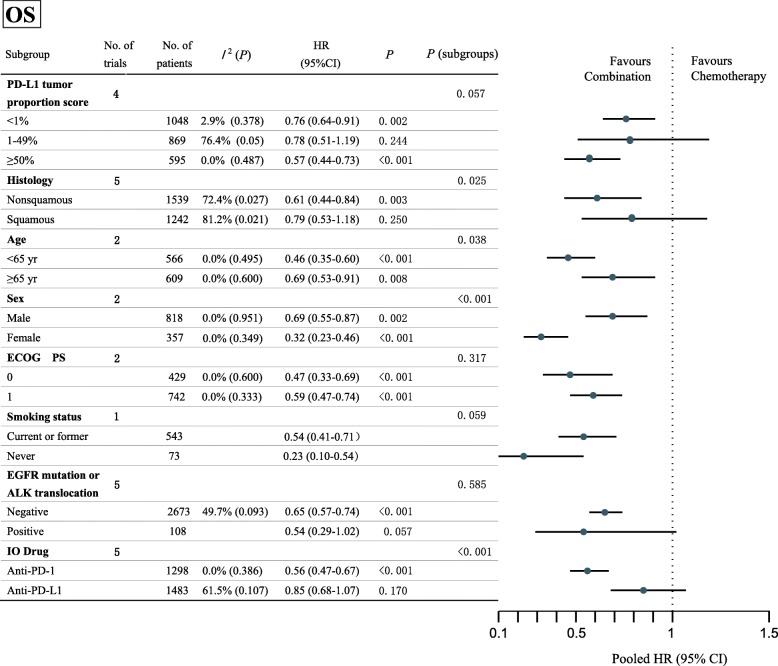
Forest plot of hazard ratios in subgroup-analyses comparing overall survival in patients who received IO-Chemotherapy vs Chemotherapy alone. The horizontal line crossing the dot represents the 95%CI of the pooled hazard ratio in each subgroup-analysis. No. of trials refers to the number of trials included in each subgroup-analysis. *I*^2^ (*P*) shows the heterogeneity in each subgroup meta-analysis. *P* (subgroups) demonstrates the significance of differences between the subgroups. HR, hazard ratio; CI, confidence interval; ECOG PS, Eastern Cooperative Oncology Group performance status; EGFR, epidermal growth factor receptor; ALK, Anaplastic lymphoma kinase; PD-1, programmed cell death 1; PD-L1, programmed cell death 1 ligand 1; IO, Immuno-oncology

The number of trials with available data for subgroup-analysis was summarized in Additional file 3: Table S4. Results of the subgroup-analyses for PFS, OS and ORR were summarized in Fig. 2, Additional file 2: Figures S3 and S4.

### Subgroup analyses by PD-L1 expression level

**Fig. 3:**
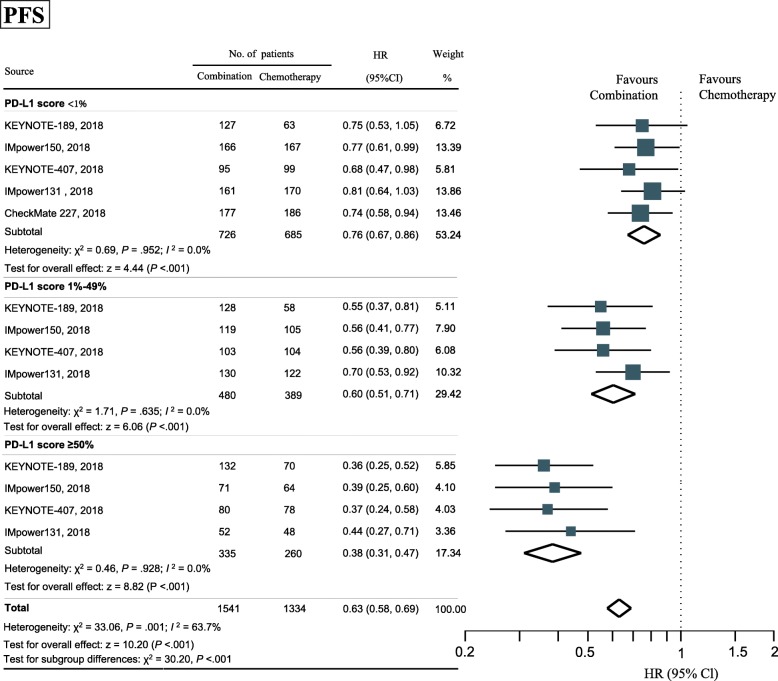
Forest plot of hazard ratios by PD-L1 expression comparing progression-free survival in patients who received IO-Chemotherapy vs Chemotherapy alone. Squares represent study-specific effect size (HR or RR). The area of square is inversely proportional to the standard error of the study and larger area indicates greater weight in the calculation of the pooled effect size. The horizontal line crossing the square represents the 95% CI. The diamonds represent the estimated overall effect, based on the meta-analysis. HR, hazard ratio; IO, Immuno-oncology; CI, confidence interval; PD-L1, programmed cell death 1 ligand 1

PD-1/PD-L1 inhibitor plus chemotherapy led to statistically longer PFS across all tested subgroups of PD-L1 expression level, including those with a PD-L1 TPS of less than 1% (HR, 0.76; 95% CI, 0.67–0.86; *P* < .001; heterogeneity, *P* = .952), a score of 1 to 49% (HR, 0.60; 95% CI, 0.51–0.71; *P* < .001; heterogeneity, *P* = .635), and a score of at least 50% (HR, 0.38; 95% CI, 0.31–0.47; *P* < .001; heterogeneity, *P* = .928) (Fig. 3). The magnitude of PFS benefit was significantly different among subgroups of PD-L1 TPS (*P* < .001).

For patients in whom the PD-L1 TPS was less than 1%, the pooled HR for OS was 0.76 (95% CI, 0.64–0.91; *P* = .002; heterogeneity, *P* = .378), compared with the HR of 0.78 (95% CI, 0.51–1.19; *P* = .244; heterogeneity, *P* = .050) in those with a score of 1 to 49% and 0.57 (95% CI, 0.44–0.73, *P* < .001; heterogeneity, *P* = .487) in those with a score of 50% or greater (Additional file 2: Figure S3). The difference of OS benefit across PD-L1 subgroups obtained a near-significant trend (*P* = .057).

The response rate was the highest in patients with a PD-L1 TPS of at least 50% (RR, 1.95; 95% CI 1.34–2.82; *P* < .001; heterogeneity, *P* = .093). In the subgroup with a score between 1 and 49%, the pooled RR was 1.39 (95% CI 0.98–1.96; *P* = .062; heterogeneity, *P* = .079). In the subgroup with a score of less than 1%, the pooled RR was 1.54 (95% CI 1.16–2.05; *P* = .003; heterogeneity, *P* = .064). There was no significant interaction between treatment effect in terms of ORR and PD-L1 expression level (*P* = .232).

### Subgroup analyses by other factors

None of the other factors predicted PFS benefit with the IO-chemotherapy combination vs. chemotherapy (Additional file 2: Figure S3), including histology (nonsquamous HR, 0.59 vs Squamous HR,0.65; interaction, *P* = .217), age (< 65 years HR, 0.60 vs ≥65 years HR,0.67; interaction, *P* = .377), sex (male HR, 0.65 vs Female HR, 0.60; interaction, *P* = .365), ECOG performance status (PS = 0 HR, 0.61 vs PS = 1 HR, 0.64; interaction, *P* = .629), smoking status (current or former HR, 0.61 vs Never HR, 0.68; interaction, *P* = .525), genomic alterations in *EGFR* or *ALK* (negative HR, 0.62 vs positive HR, 0.59; interaction, *P* = .860), and type of IO drug (anti-PD-1 HR, 0.58 vs anti-PD-L1, 0.65; interaction, *P* = .179).

However, there were several other factors that could predict OS and ORR benefit from the IO-chemotherapy over chemotherapy alone (Fig. 2 and Additional file 2: Figure S4). Patients with nonsquamous histology (nonsquamous HR, 0.61 vs squamous HR, 0.79; interaction, *P* = .025), younger age (< 65 years HR, 0.46 vs ≥65 years HR, 0.69; interaction, *P* = .038), who were females (female HR, 0.32 vs male HR, 0.69; interaction, *P* < .001), and who received anti-PD-1 antibody (anti-PD-1 antibody HR, 0.56 vs anti-PD-L1 antibody HR, 0.85; interaction, *P* < .001) might receive more OS benefit from the combination therapy. And patients with anti-PD-1 drug might have a higher objective response rate from the combination therapy than those with anti-PD-L1 antibody (anti-PD-1 RR, 1.81 vs anti-PD-L1 RR 1.27; interaction, *P* < .001).

### Sensitivity analyses

To evaluate the robustness of the combined outcomes, we carried out sensitivity analyses by omitting specific studies or altering statistical models. The results showed that the overall estimates remained consistent across these analyses (Additional file 2: Figure S5; Additional file 3: Tables S5 and S6).

### Safety analyses

**Fig. 4:**
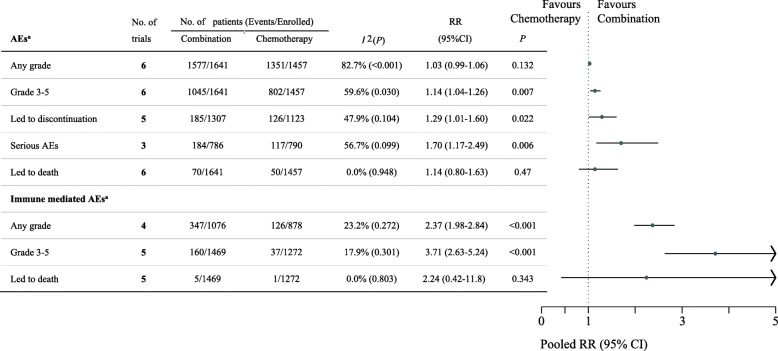
Forest plot of risk ratios comparing treatment-related adverse events in patients who received IO-Chemotherapy vs Chemotherapy alone. The horizontal line crossing the dot represents the 95%CI of the pooled risk ratio in each subgroup-analysis. No. of trials refers to the number of trials included in each subgroup-analysis. *I*^2^ (*P*) shows the heterogeneity in each subgroup meta-analysis. ^a^Data provided in KEYNOTE-189 and KEYNOTE-407 were all-cause adverse events, regardless of attribution to any treatment. CI, confidence interval; RR, risk ratio; AEs, adverse events; IO, Immuno-oncology

Safety analyses were conducted in patients who had received at least one dose of the study drug (Table 1). The median or mean duration of treatment is summarized in Additional file 3: Table S3, and the number of patients included for each safety analysis is presented in Fig. 4. The pooled results demonstrated that IO-chemotherapy combination was significantly associated with higher frequency of treatment-related AEs of grade 3 or more severity (pooled RR 1.14, 95% CI 1.04–1.26, *P* = .007), AEs leading to treatment discontinuation (pooled RR 1.29, 95% CI 1.01–1.60, *P* = .022), treatment-related serious AEs (pooled RR 1.70, 95% CI 1.17–2.49, *P* = .006), immune mediated AEs of any grade (pooled RR 2.37, 95% CI 1.98–2.84, *P* < .001), and immune mediated AEs of grade 3 or more severity (pooled RR 3.71, 95% CI 2.63–5.24, *P* < .001). However, there was no significant difference in the frequency of AEs of any grade (pooled RR 1.03, 95% CI 0.99–1.06, *P* = .132), death attributed to treatment (pooled RR 1.14, 95% CI 0.80–1.63, *P* = .47) and immune mediated AEs leading to death (pooled RR 2.24, 95% CI 0.42–11.8, *P* = .343).

## Discussion

To the best of our knowledge, this is the first meta-analysis that focuses on investigating the association between PD-1/PD-L1 inhibitor plus chemotherapy versus chemotherapy and treatment outcomes in patients with advanced NSCLC. The analysis demonstrates that PD-1/PD-L1 inhibitor plus chemotherapy is statistically associated with a 38% reduction in the risk of disease progression, a 32% reduction in the risk of death, and 1.6 times the probability of achieving an objective response compared with standard chemotherapy for first-line treatment of advanced NSCLC, though at the expense of increased treatment-related adverse events. The significant predictor(s) for treatment benefit with IO-chemotherapy combination vs chemotherapy were PD-L1 expression for PFS; IO drug type for ORR; histology, age, gender, and IO drug type for OS.

One plausible point about IO combination therapy is its potential for increasing the percentage responders who otherwise do not respond to monotherapy. In the KEYNOTE-024 and -042 studies, pembrolizumab leads to statistically significant and clinically meaningful improvement of OS in patients with PD-L1 TPS of 50% or greater (HR, 0.63 and 0.69, respectively) [4, 21]; however there is no significant OS difference in patients with PD-L1 TPS of 1–49% (HR, 0.92). This is clinically relevant because patients with high PD-L1 expression represent a minority of those with NSCLC [22]. And less than one half of patients with advanced NSCLC ever receive second-line therapy due to rapid deterioration during disease progression [22, 23]. Therefore, maximizing the chance of response to first-line treatment is important. In the present study, we found that PD-1/PD-L1 inhibitor plus chemotherapy led to improved survival across all tested subgroups of patients according to PD-L1 expression, including patients with low or negative PD-L1 expression. These findings in unselected patients are particularly relevant because the majority of patients have tumors with low, negative, or undetectable PD-L1 expression [22]. Furthermore, there are multiple challenges regarding using PD-L1 as a predictive marker of immunotherapy, including the intratumoral heterogeneity and interassay discordance of PD-L1 expression [24–26]. Nevertheless, the greatest benefit was observed in the subgroup with a PD-L1 TPS of 50% or more (HR for OS, 0.57). This finding also raises a clinically important question whether PD-1/PD-L1 inhibitor plus chemotherapy has greater efficacy than the single agent PD-1/PD-L1 inhibitor in these patients, which needs to be directly compared in randomized studies.

In subgroup analysis, there was a significant difference in treatment benefits between PD-1 inhibitor and PD-L1 inhibitor when combined with chemotherapy (HR for OS, 0.56 vs 0.85; interaction, *P* < .001; RR for ORR, 1.81 vs 1.27; interaction, *P* < .001). Similarly, a previous network meta-analysis demonstrates that the probability of treatment ranking was higher with PD-1 inhibitor than PD-L1 inhibitor for previously treated NSCLC [27]. One possible explanation is that anti-PD-L1 antibody spares the interaction between PD-1 and PD-L2 [28]. However, cautions should be exercised in deciding which drug class is preferred due to the lack of head-to-head clinical trials.

Our study also reveals a statistically significant interaction between histology and OS benefit (nonsquamous HR, 0.61 vs squamous HR, 0.79; interaction *P* = .025). This seems consistent with the previous belief that patients with squamous NSCLC receive less benefit from checkpoint inhibitors [29]. Nevertheless, this subgroup analysis does not deny the benefits of IO-chemotherapy for squamous NSCLC. Rather, exploratory biomarker analysis and extended follow-up are needed to fully evaluate the role of IO-chemotherapy in patients with squamous NSCLC, in whom treatment options have been very limited for decades and the prognosis remains poor.

Interestingly, a recent meta-analysis demonstrates that the relative benefit of immunotherapy (predominantly single agent) is greater in male cancer patients than in female patients [30]. In contrast with this report, our study demonstrates greater OS benefit with PD-1/PD-L1 inhibitor plus chemotherapy for female patients than for male patients (female HR, 0.32 vs male HR, 0.69; interaction *P* < .001). However, it should be noted that the subgroup analysis of OS regarding gender was only available from the pembrolizumab studies, KEYNOTE-189 [6] and KEYNOTE-407 [19]. Therefore, it remains controversy whether the magnitude of OS benefit with immune checkpoint inhibitors are sex-dependent. Finally, whether the relatively lower efficacy of immunotherapy in female patients could be reversed by applying combinatorial treatment strategy as evident in this analysis warrants further investigation.

We also showed that IO-chemotherapy significantly improved OS both in patients < 65 years old (HR 0.46; *P* < .001) and ≥ 65 years old (HR 0.69; *P* = .008), with greater benefit in in younger patients (interaction *P* = .038). This finding is different from previous meta-analyses which revealed similar efficacy with immune checkpoint inhibitor monotherapy in patients younger vs. older than 65 years old [31, 32]. These results are clinically relevant because a majority of lung cancer patients were diagnosed at older ages and were often with poor performance status in real world [33]. Considering the higher frequency of treatment-related adverse events with IO-chemotherapy, it remains an open question how to tailor the treatment for older patients to optimize clinical outcomes in the era of immunotherapy.

This meta-analysis further unfolded that smoking history, *EGFR* mutation or *ALK* rearrangement, and PS 0 or 1 were not predictive of OS benefit with IO-chemotherapy vs chemotherapy. Typically, patients with *EGFR* or *ALK* genomic alterations receive little OS advantage with the single agent PD-1/PD-L1 inhibitor [34]. Despite the high PD-L1 expression in oncogene-addicted tumors [35, 36], they are associated with a high frequency of inactive tumor-infiltrating lymphocytes [37], low mutation load [38], and weak immunogenicity [39]. These factors are hypothesized to account for the inferior efficacy of immunotherapy in patients with *EGFR*- or *ALK*-driven NSCLC. However, the IMpower 150 study showed that combination therapy with atezolizumab, bevacizumab and chemotherapy significantly improved survival in these patients [15]. It remains unclear whether it is the addition of chemotherapy or anti-angiogenesis agent or both that reverse the “cold” immune microenvironment in oncogene-driven NSCLC.

Regarding the safety profiles, the addition of PD-1/PD-L1 inhibitor to chemotherapy was significantly associated with increased risk of developing AEs of grade 3 or worse severity, treatment-related drug discontinuation and serious AEs. However, the frequency of deaths attributed to treatment was similar between the both groups. Immune-mediated adverse events were more frequently observed in the IO-chemotherapy group, with frequency and severity consistent with those noted in the PD-1/PD-L1 inhibitor monotherapy [40].

A strength of this work is the quality of evidence available and used in the meta-analysis. Source data were obtained from six randomised controlled trials that involved over 3000 patients. Thus, the meta-analysis could overcome the problem of inadequate power of each individual trial by pooling data together. Albeit the strength above, we encountered several limitations during this study. First of all, our meta-analysis relies on published results rather than on individual patients’ data. Therefore, the results from subgroup analysis remain inconclusive but merely suggestive. The optimal clinical and molecular predictors of benefit from IO-chemotherapy remains to be elucidated. Secondly, the clinical trials included in this meta-analysis enrolled highly selective patients, e.g. patients with good performance status, sufficient organ functions and no comorbidities like autoimmune disease. Therefore, we were unable to explore the effect of these regimens in patients who were ineligible for clinical trials. And these patients represent a large proportion in real-world clinical practice. Finally, the OS data from the included trials were not mature enough. An update meta-analysis with final OS data will be important in the future. Yet, this meta-analysis established the definite benefit of IO-chemotherapy vs chemotherapy in terms of PFS and ORR in the first line setting, which might serve as moderate surrogate endpoints for OS [41].

In conclusion, PD-1/PD-L1 inhibitor plus chemotherapy, compared with chemotherapy, significantly prolonged PFS and OS in first-line of treatment for advanced NSCLC, irrespective of PD-L1 expression level. Future studies are needed to explore reliable predictors of treatment efficacy and to determine which chemotherapeutic modality will improve patient’s survival in combination with PD-1/PD-L1 inhibitor. Finally, the trade-off between benefits and risk of side effects as well as treatment costs should be considered in clinical practice.

## Additional files

Additional file 1: **Supplementary Method.** Search strategies for PubMed, EMBASE, and Cochrane database. (PDF 314 kb)

Additional file 2 **Figure S1.** Trial selection process. **Figure S2.** Funnel plot comparing hazard ratios for (A) progression-free survival and (B) overall survival, and risk ratios for (C) objective response rate. Each study’s effect estimate plotted against its standard error. The outer dashed lines represent the confidence interval boundary within which 95% of studies are expected to lie in the absence of bias or heterogeneity. The solid vertical line represents the summary treatment effect. **Figure S3.** Forest plot of hazard ratios in subgroup-analyses comparing progression-free survival in patients who received IO-Chemotherapy vs Chemotherapy alone. The horizontal line crossing the dot represents the 95%CI of the pooled hazard ratio in each subgroup-analysis. No. of trials refers to the number of trials included in each subgroup-analysis. *I*^2^ (*P*) shows the heterogeneity in each subgroup meta-analysis. *P* (subgroups) demonstrates the significance of differences between the subgroups. CI, confidence interval; ECOG PS, ECOG performance-status score; and IO, Immuno-oncology. **Figure S4.** Forest plot of risk ratios in subgroup-analyses comparing objective response rate in patients who received IO-Chemotherapy vs Chemotherapy alone. The horizontal line crossing the dot represents the 95%CI of the pooled risk ratio in each subgroup-analysis. No. of trials refers to the number of trials included in each subgroup-analysis. *I*^2^ (*P*) shows the heterogeneity in each subgroup meta-analysis. *P* (subgroups) demonstrates the significance of differences between the subgroups. IO, Immuno-oncology. **Figure S5.** Sensitivity analyses of progression-free survival (PFS), overall survival (OS), objective response rate (ORR) by repeating the pooled analyses with one study omitted at a time. (PDF 609 kb)

Additional file 3 **Table S1.** Quality assessment: risk of bias by Cochrane Collaboration’s tool. **Table S2.** Additional characteristics of patients comparing IO-Chemotherapy with Chemotherapy in Included trials. **Table S3.** Main outcomes of the included trials**. Table S4.** Summary of the data status for subgroup-analyses among the included trials. **Table S5.** Summary of sensitivity analyses results using both fixed-effects and random-effects models. **Table S6.** Summary of sensitivity analyses after removing studies that were only available from conference presentation. (PDF 982 kb)

## Abbreviations

AACR: American Association for Cancer Research
AEs: Adverse events
ASCO: American Society of Clinical Oncology
CI: Confidence interval
ECOG PS: ECOG performance-status score
ESMO: European Society of Medical Oncology
HLA: Human leucocyte antigen
HR: Hazard ratio
IO: Immuno-oncology
NSCLC: Non-small-cell lung carcinoma
ORR: Objective response rate
OS: Overall survival
PD-1: Programmed cell death 1
PD-L1: Programmed cell death 1 ligand 1
PFS: Progression-free survival
RR: Risk ratio
TPS: Tumor proportion score tumor proportion score
WCLC: World Conference on Lung Cancer
