# Supplementary material for: Immune-checkpoint inhibitor plus chemotherapy versus conventional chemotherapy for first-line treatment in advanced non-small cell lung carcinoma: a systematic review and meta-analysis
Source: J Immunother Cancer. 2018 Dec 22;6:155. doi: 10.1186/s40425-018-0477-9 (PMC6303974; doi:10.1186/s40425-018-0477-9)
Supplement: Supplementary file 1 — Supplementary Method. Search strategies for PubMed, EMBASE, and Cochrane database. (PDF 314 kb) [file 40425_2018_477_MOESM1_ESM.pdf]

Supplementary Method: Search strategies for PubMed, EMBASE, and Cochrane database

Pubmed: 524 results

(("pembrolizumab" [Supplementary Concept] OR "lambrolizumab"[Title/Abstract] OR "Keytruda"[Title/Abstract] OR "MK-3475"[Title/Abstract] OR "nivolumab"[Supplementary Concept] OR "MDX-1106"[Title/Abstract] OR "ONO-4538"[Title/Abstract] OR "BMS-936558"[Title/Abstract] OR "Opdivo"[Title/Abstract] OR "atezolizumab"[Supplementary Concept] OR "anti-PDL1"[Title/Abstract] OR "MPDL3280A"[Title/Abstract] OR "Tecentriq"[Title/Abstract] OR "RG7446"[Title/Abstract] OR "RG-7446"[Title/Abstract] OR "Durvalumab" [Title/Abstract] OR "anti-PD1"[Title/Abstract] OR "PD-1"[Title/Abstract] OR "Programmed Death 1"[Title/Abstract] OR "Programmed Cell Death 1 Receptor"[Title/Abstract] OR "PD 1"[Title/Abstract] OR "PD1"[Title/Abstract] OR "Programmed Death-Ligand 1"[Title/Abstract] OR "PD-L1"[Title/Abstract] OR "programmed cell death 1 ligand 1 protein"[Title/Abstract] OR "PD L1"[Title/Abstract] OR "PDL1"[Title/Abstract])) AND (((((lung[Title/Abstract]) AND (((((NSCLC[Title/Abstract]) OR "Non Small Cell"[Title/Abstract]) OR "Non-Small-Cell"[Title/Abstract]) OR "Non-Small Cell"[Title/Abstract]) OR "Non-Small Cell"[Title/Abstract]))) OR "Carcinoma, Non-Small-Cell Lung"[Mesh]) AND (("clinical trials as topic"[MeSH Terms] OR "trial"[Title/Abstract] OR "study"[Title/Abstract]))

EMbase: 283 results

(‘pembrolizumab’/exp OR ‘lambrolizumab’:ab,ti OR ‘Keytruda’:ab,ti OR ‘MK-3475’:ab,ti OR ‘nivolumab’/exp OR ‘MDX-1106’:ab,ti OR ‘ONO-4538’:ab,ti OR ‘BMS-936558’:ab,ti OR ‘Opdivo’:ab,ti OR ‘atezolizumab’/exp OR ‘anti-PDL1’:ab,ti OR ‘MPDL3280A’:ab,ti OR ‘Tecentriq’:ab,ti OR ‘RG7446’:ab,ti OR ‘RG-7446’:ab,ti OR ‘Durvalumab’:ab,ti OR ‘anti-PD1’:ab,ti OR ‘PD-1’:ab,ti OR ‘Programmed Death 1’:ab,ti OR ‘Programmed Cell Death 1 Receptor’:ab,ti OR ‘PD 1’:ab,ti OR ‘PD1’:ab,ti OR ‘Programmed Death-Ligand 1’:ab,ti OR ‘PD-L1’:ab,ti OR ‘programmed cell death 1 ligand 1 protein’:ab,ti OR ‘PD L1’:ab,ti OR ‘PDL1’:ab,ti) AND (('non small cell lung cancer'/exp OR ('lung':ab,ti AND (‘NSCLC’:ab,ti OR ‘Non Small Cell’:ab,ti OR ‘Non-Small-Cell’:ab,ti OR ‘Non-Small Cell’:ab,ti OR ‘Non-Small Cell’:ab,ti))) AND (‘randomized controlled trial’/exp))

Cochrane: 518 results, 508 trials

#1 MeSH descriptor: [Carcinoma, Non-Small-Cell Lung] explode all trees

#2 “lung” AND ("Non Small Cell" OR "Non-Small Cell" OR "Non-Small-Cell")

#3 "pembrolizumab" or "lambrolizumab" or "Keytruda" or "MK-3475" or "nivolumab" or "MDX-1106" or "ONO-4538" or "BMS-936558" or "Opdivo" or "atezolizumab" or "anti-PDL1" or "MPDL3280A" or "Tecentriq" or "RG7446" or "RG-7446" or "Durvalumab" or "anti-PD1" or "PD-1" or "Programmed Death 1" or "Programmed Cell Death 1 Receptor" or "PD 1" or "PD1" or "Programmed Death-Ligand 1" or "PD-L1" or "programmed cell death 1 ligand 1 protein" or "PD L1" or "PDL1"

4 (#1 OR #2) AND #3
