# Supplementary material for: Immune-checkpoint inhibitor plus chemotherapy versus conventional chemotherapy for first-line treatment in advanced non-small cell lung carcinoma: a systematic review and meta-analysis
Source: J Immunother Cancer. 2018 Dec 22;6:155. doi: 10.1186/s40425-018-0477-9 (PMC6303974; doi:10.1186/s40425-018-0477-9)

Supplementary Figure 1. Trial Selection Process

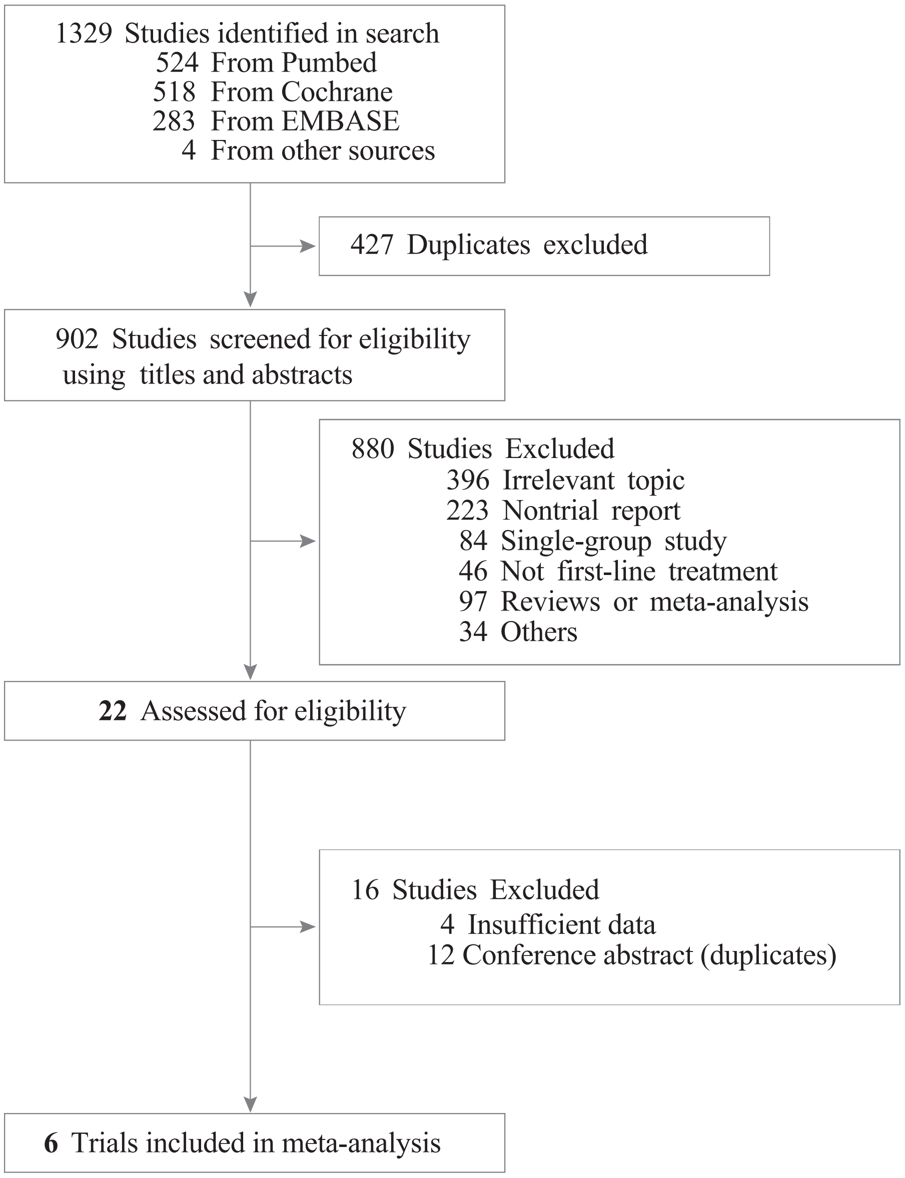

Supplementary Figure 2. Funnel Plot comparing Hazard Ratios for Progression-free Survival and Overall Survival, and Risk Ratios for Objective Response Rate

A Progression-free Survival

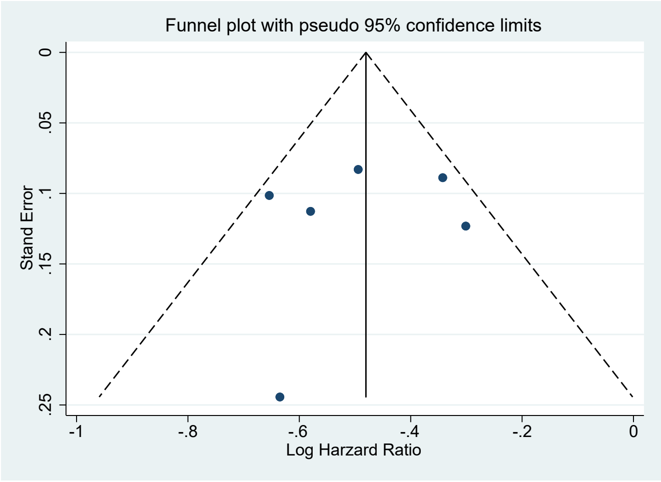

B Overall Survival

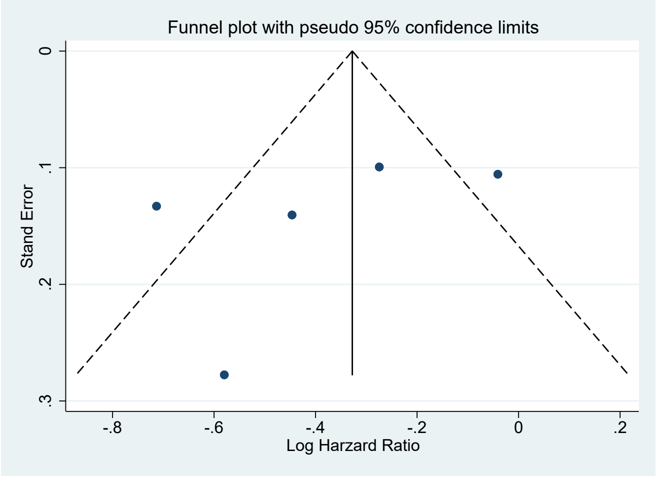

C Objective Response Rate

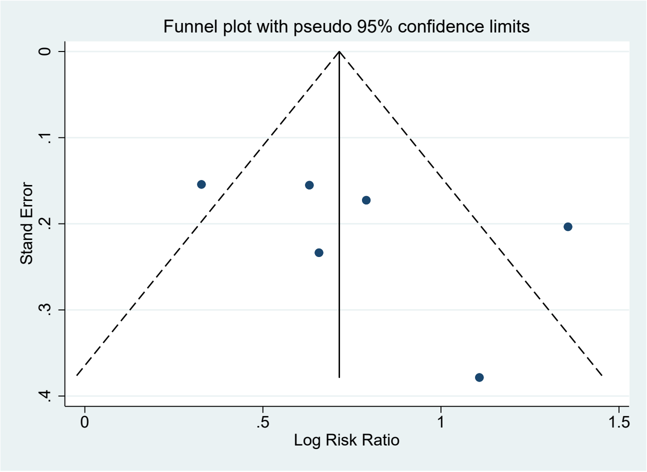

Supplementary Figure 3. Forest Plot of Hazard Ratios in Subgroup-analyses Comparing Progression-free Survival in Patients Who Received IO-Chemotherapy vs Chemotherapy alone

Subgroup	No. of trials	No. of patients	I^2 (P)	HR (95%CI)	P	P (subgroups)
**PD-L1 tumor proportion score**	5					<0. 001
<1%		1411	0.0% (0.952)	0.76 (0.67-0.86)	<0. 001	
1-49%		869	0.0% (0.635)	0.60 (0.51-0.71)	<0. 001	
≥50%		595	0.0% (0.928)	0.38 (0.31-0.47)	<0. 001	
**Histology**	6					0. 217
Nonsquamous		1813	43.1% (0.135)	0.59 (0.52-0.66)	<0. 001	
Squamous		1331	29.2% (0.243)	0.65 (0.58-0.74)	<0. 001	
**Age**	4					0. 377
<65 yr		1202	69.6% (0.020)	0.60 (0.47-0.77)	<0. 001	
≥65 yr		1146	21.5% (0.272)	0.67 (0.57-0.79)	<0. 001	
**Sex**	4					0. 365
Male		1600	12.7% (0.329)	0.65 (0.58-0.74)	<0. 001	
Female		754	66.7% (0.029)	0.60 (0.44-0.81)	<0. 001	
**ECOG PS**	4					0. 629
0		889	43.7% (0.150)	0.61 (0.52-0.72)	<0. 001	
1		1450	0.0% (0.624)	0.64 (0.57-0.72)	<0. 001	
**Smoking status**	3					0. 525
Current or former		1754	50.0% (0.138）	0.61 (0.55-0.69）	<0. 001	
Never		236	27.0%（0.254）	0.68 0.50-0.93)	0. 015	
**EGFR mutation or ALK translocation**	6					0. 860
Negative		3036	47.0% (0.093)	0.62 (0.56-0.67)	<0. 001	
Positive		108		0.59 (0.37-0.94)	0. 027	
**IO Drug**	6					0. 179
Anti-PD-1		1661	43.4% (0.151)	0.58 (0.52-0.66)	<0. 001	
Anti-PD-L1		1483	35.8% (0.212)	0.65 (0.58-0.74)	<0. 001	

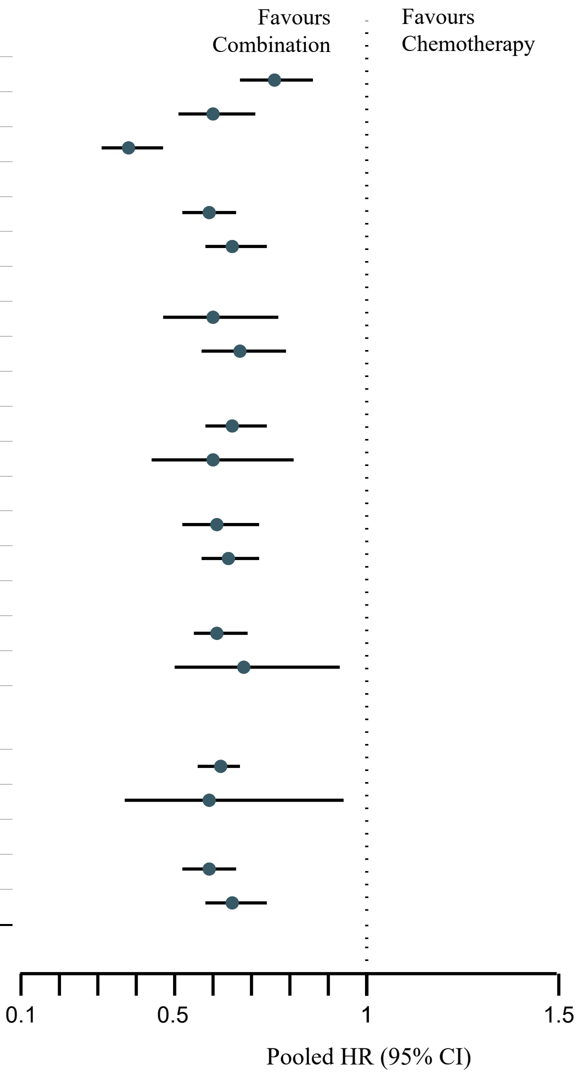

Supplementary Figure 4. Forest Plot of Risk Ratios in Subgroup-analyses Comparing Objective Response Rate in Patients Who Received IO-Chemotherapy vs Chemotherapy Alone

Subgroup	No. of trials	No. of patients	I^2 (P)	RR (95%CI)	P	P (subgroups)
**PD-L1 tumor proportion score**	5					0.232
<1%		1071	55.0% (0.064)	1.54 (1.16-2.05)	0.003	
1-49%		572	55.8% (0.079)	1.39 (0.98-1.96)	0.062	
≥50%		422	53.2% (0.093)	1.95 (1.34-2.82)	<0.001	
**Histology**	5					0.168
Nonsquamous		1431	88.5% (<0.001)	1.81 (1.13-2.91)	0.013	
Squamous		1242	70.8% (0.064)	1.34 (1.07-1.68)	0.011	
**IO Drug**	6					<0.001
Anti-PD-1		1661	66.8% (0.029)	1.81 (1.40-2.34)	<0.001	
Anti-PD-L1		1375	0.0% (0.366)	1.27 (1.14-1.41)	<0.001	

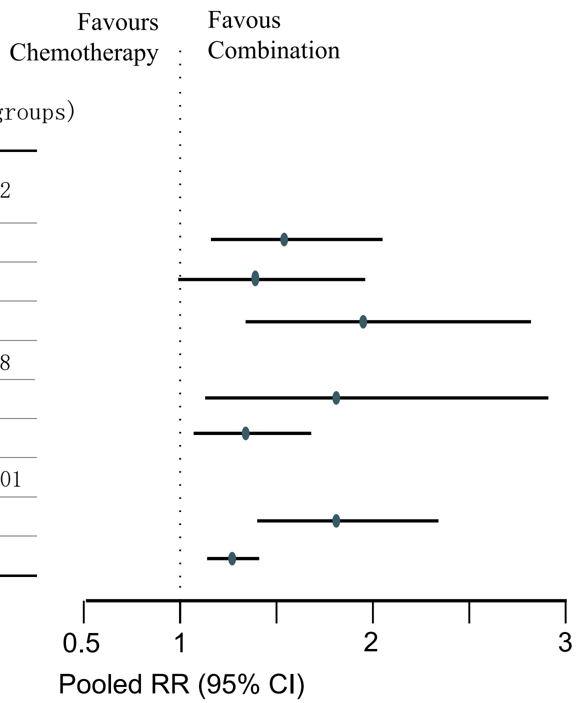

Supplementary figure 5. Sensitivity analyses of progression-free survival (PFS), overall survival (OS), objective response rate (ORR) by repeating the pooled analyses with one study omitted at a time.

A. PFS

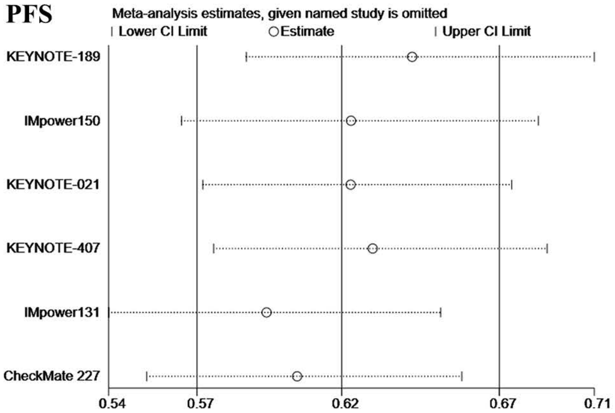

B. OS

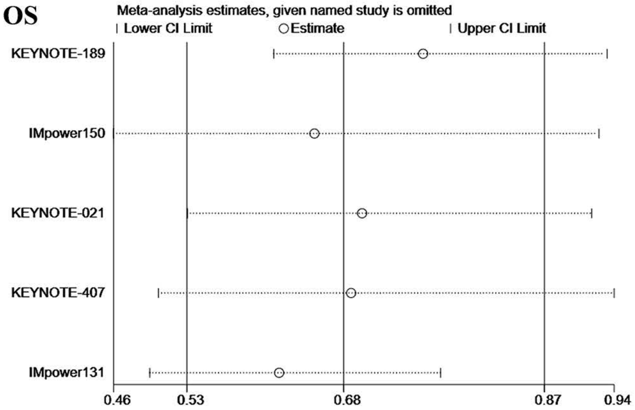

C. ORR

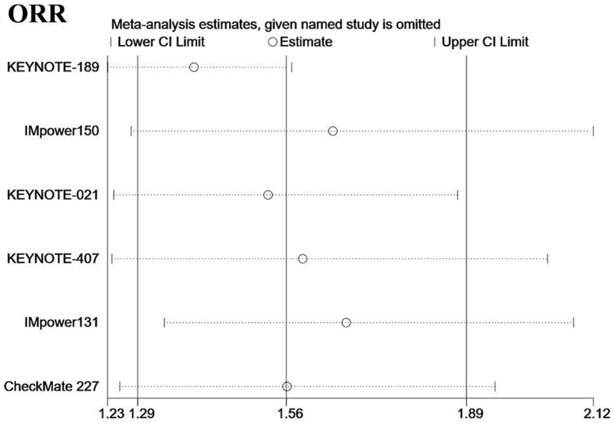

Supplement: Supplementary file 2 — Figure S1. Trial selection process. Figure S2. Funnel plot comparing hazard ratios for (A) progression-free survival and (B) overall survival, and risk ratios for (C) objective response rate. Each study’s effect estimate plotted against its standard error. The outer dashed lines represent the confidence interval boundary within which 95% of studies are expected to lie in the absence of bias or heterogeneity. The solid vertical line represents the summary treatment effect. Figure S3. Forest plot of hazard ratios in subgroup-analyses comparing progression-free survival in patients who received IO-Chemotherapy vs Chemotherapy alone. The horizontal line crossing the dot represents the 95%CI of the pooled hazard ratio in each subgroup-analysis. No. of trials refers to the number of trials included in each subgroup-analysis. I2 (P) shows the heterogeneity in each subgroup meta-analysis. P (subgroups) demonstrates the significance of differences between the subgroups. CI, confidence interval; ECOG PS, ECOG performance-status score; and IO, Immuno-oncology. Figure S4. Forest plot of risk ratios in subgroup-analyses comparing objective response rate in patients who received IO-Chemotherapy vs Chemotherapy alone. The horizontal line crossing the dot represents the 95%CI of the pooled risk ratio in each subgroup-analysis. No. of trials refers to the number of trials included in each subgroup-analysis. I2 (P) shows the heterogeneity in each subgroup meta-analysis. P (subgroups) demonstrates the significance of differences between the subgroups. IO, Immuno-oncology. Figure S5. Sensitivity analyses of progression-free survival (PFS), overall survival (OS), objective response rate (ORR) by repeating the pooled analyses with one study omitted at a time. (PDF 609 kb) [file 40425_2018_477_MOESM2_ESM.pdf]
