# Supplementary material for: Immune-checkpoint inhibitor plus chemotherapy versus conventional chemotherapy for first-line treatment in advanced non-small cell lung carcinoma: a systematic review and meta-analysis
Source: J Immunother Cancer. 2018 Dec 22;6:155. doi: 10.1186/s40425-018-0477-9 (PMC6303974; doi:10.1186/s40425-018-0477-9)
Supplement: Supplementary file 3 — Table S1. Quality assessment: risk of bias by Cochrane Collaboration’s tool. Table S2. Additional characteristics of patients comparing IO-Chemotherapy with Chemotherapy in Included trials. Table S3. Main outcomes of the included trials. Table S4. Summary of the data status for subgroup-analyses among the included trials. Table S5. Summary of sensitivity analyses results using both fixed-effects and random-effects models. Table S6. Summary of sensitivity analyses after removing studies that were only available from conference presentation. (PDF 982 kb) [file 40425_2018_477_MOESM3_ESM.pdf]

Supplementary Table 1. Quality assessment: risk of bias by Cochrane Collaboration's tool

Trial	Sequence generation	Allocation concealment	Blinding	Incomplete outcome data	Selective reporting	Other source of bias
KEYNOTE-189, 2018	Adequate	Adequate (Central allocation)	Adequate (Independent radiologic review)	Adequate	Adequate	
IMpower150, 2018	Adequate	Adequate (Central allocation)	Adequate (Independent radiologic review)	Adequate	Adequate	
KEYNOTE-021, 2016 and 2018	Adequate	Adequate (Central allocation)	Adequate (Independent radiologic review)	Adequate	Adequate	
KEYNOTE-407, 2018	Adequate	Adequate (Central allocation)	Adequate (Independent radiologic review)	Adequate	Adequate	Data from the abstract and the presentation slides
IMpower131, 2018	Adequate	Adequate (Central allocation)	Adequate (Independent radiologic review)	Adequate	Adequate	Data from the abstract and the presentation slides
CheckMate 227, 2018	Adequate	Adequate (Central allocation)	Adequate (Independent radiologic review)	Adequate	Inadequate (overall survival was not reported yet)	Data from the abstract and the presentation slides

Supplementary Table 2. Additional characteristics of patients comparing IO-Chemotherapy with Chemotherapy in included trials

Source	Design	Chemotherapy Drug	Disease stage	Enrolment time	Primary end point	Secondary end point	Positive[a] EGFR mutation (n (%))	Positive[a] ALK mutation (n (%))	PD-L1 staining antibody clone
KEYNOTE-189, 2018	Phase 3, double-blind, Crossover permitted	AP or AC<br>i) cisplatin (75 mg/m^2 Q3W) or carboplatin (6mg/ml/min Q3W）<br>ii) pemetrexed (500 mg/m^2 Q3W)	IV	2016-2017	PFS、OS	ORR、DOR、safety	0	0	22C3
IMpower150, 2018	Phase 3, open-lable, Crossover not permitted	BCP<br>i) carboplatin (6mg/ml/min Q3W）<br>ii)paclitaxel (200 mg/m^2 Q3W)<br>iii)bevacizumab(15 mg/kg Q3W)	IV	2015-2016	PFS、OS in the wild-type	PFS、OS in ITT、ORR、DOR、safety	35(8.8) vs 45(11.3)	13 (3.2) vs 21 (5.2)	SP142
KEYNOTE-021, 2016 and 2018	Phase 2, open-lable, Crossover permitted	AC<br>i) carboplatin (5mg/ml/min Q3W）<br>ii) pemetrexed (500 mg/m^2 Q3W)	IIIB or IV	2014-2016	ORR	PFS、OS、DOR、safety	0	0	22C3
KEYNOTE-407, 2018	Phase 3, double-blind, Crossover permitted	PC<br>i) carboplatin (6mg/ml/min Q3W）<br>ii) paclitaxel（200mg/m^2 Q3W）or nab-paclitaxel（100mg/m^2 Q1W）	IV	2016-2018	PFS、OS	ORR、DOR、safety	NR	NR	22C3
IMpower131, 2018	Phase 3, open-lable, Crossover not permitted	PC<br>i) carboplatin (6mg/ml/min Q3W）<br>ii) nab-paclitaxel（100mg/m^2 Q1W）	IV	2015-2017	PFS、OS	ORR、DOR、safety	NR	NR	SP142
CheckMate 227, 2018	Phase 3, open-lable	Non-squamous:pemetrexed+cisplatin or carboplatin Q3W;<br>Squamous:gemcitabine+cisplatin or carboplatin Q3W	IV	2015-	PFS、OS	ORR、DOR、safety	0	0	28-8

[a] Data presented as "IO-chemotherapy group vs chemotherapy group".

Abbreviations: IO, immuno-oncology; NR, not reported; PFS, progression-free survival; OS, overall survival; ORR, objective response rate; DOR, duration of response.

Supplementary Table 3. Main outcomes of the included trials

Source	Median Follow-up (months)	PFS (months)[a]	OS (months)[a]	ORR (%)[a]	DOR[a] (months)	Duration[a] of treatment (months)
KEYNOTE-189, 2018	10.5	8.8 vs 4.9	Not Reached vs 11.3	48% vs 19%	11.2 vs 7.8	7.4 vs 5.4[b]
IMpower150, 2018	20	8.3 vs 6.8	19.2 vs. 14.7	56% vs 41%	11.5 vs 6.0	8.2 vs 5.1[c]
KEYNOTE-021, 2016 and 2018	23.9	24.0 vs 9.3	Not Reached vs 21.1	57% vs 30%	Not Reached vs 16.2	8.0 vs 4.9[b]
KEYNOTE-407, 2018	7.8	6.4 vs 4.8	15.9 vs 11.3	58% vs 38%	7.7 vs 4.8	6.3 vs 4.7[c]
IMpower131, 2018	17.1	6.3 vs 5.6	14.0 vs 13.9	49% vs 41%	7.2 vs 5.2	6.7 vs 2.8[c]
CheckMate 227, 2018	11.2	5.6 vs 4.7	NR	37% vs 23%	7.2 vs 4.7	8.5 cycles[c] vs 4-7 cycles

[a] Data presented as "IO-chemotherapy group vs chemotherapy group".

[b] mean duration of treatment

[c] median duration of treatment

Abbreviations: PFS, progression-free survival; OS, overall survival; ORR, objective response rate; DOR, duration of response.

Supplementary Table 4. Summary of the data status for subgroup-analyses among the included trials

Source	PD-L1 tumor proportion score			Histology			Age			Sex			ECOG PS			Smoking status			EGFR mutation or ALK translocation			IO Drug		
	PFS	OS	ORR	PFS	OS	ORR	PFS	OS	ORR	PFS	OS	ORR	PFS	OS	ORR	PFS	OS	ORR	PFS	OS	ORR	PFS	OS	ORR
KEYNOTE-189, 2018	Y	Y	Y	Y	Y	Y	Y	Y	N	Y	Y	N	Y	Y	N	Y	Y	N	Y	Y	N	Y	Y	Y
IMpower150, 2018	Y	Y	Y	Y	Y	Y	Y	N	N	Y	N	N	Y	N	N	Y	N	N	Y	Y	N	Y	Y	Y
KEYNOTE-021, 2016 and 2018	N	N	Y	Y	Y	Y	N	N	N	N	N	N	N	N	N	N	N	N	Y	Y	N	Y	Y	Y
KEYNOTE-407, 2018	Y	Y	N	Y	Y	Y	N	Y	N	N	Y	N	N	Y	N	N	N	N	Y	Y	N	Y	Y	Y
IMpower131, 2018	Y	Y	Y	Y	Y	Y	Y	N	N	Y	N	N	Y	N	N	Y	N	N	Y	Y	N	Y	Y	Y
CheckMate 227, 2018	Y	N	Y	Y	N	N	Y	N	N	Y	N	N	Y	N	N	N	N	N	Y	N	N	Y	N	Y
Trials Enrolled (n)	5	4	5	6	5	5	4	2	0	4	2	0	4	2	0	3	1	0	6	5	0	6	5	6

Abbreviations: Y, data available; N, data not available; PFS, progression-free survival; OS, overall survival; ORR, objective response rate; ECOG PS, ECOG performance-status score； IO, immuno-oncology.

Supplementary Table 5. Summary of sensitivity analyses results using both fixed-effects and random-effects models.

Outcome	Subgroup	No. of Studies	HR/RR	LL	UL	Effect Size Z	*P*-value	Heterogeneity *P*-value	I^2
PFS	Fixed-Effects Model	6	0.62	0.57	0.67	11.06	<0.001	0.123	42.3%
	Random-Effects Model	6	0.62	0.55	0.69	8.12	<0.001	0.123	42.3%
OS	Fixed-Effects Model	5	0.72	0.65	0.81	5.78	<0.001	<0.001	77.3%
	Random-Effects Model	5	0.68	0.53	0.87	3.04	0.002	<0.001	77.3%
ORR	Random-Effects Model	6	1.48	1.36	1.62	9.19	<0.001	<0.001	77.6%
	Fixed-Effects Model	6	1.56	1.29	1.89	4.52	<0.001	<0.001	77.6%

PFS, progression-free survival; OS, overall survival; ORR, objective response rate; HR, hazard ratio; RR, relative risk; LL, lower limit; UL, upper limit

Supplementary Table 6. Summary of sensitivity analyses after removing studies that were only available from conference presentation.

Outcome	Subgroup	No. of Studies	HR/RR	LL	UL	Effect Size Z	*P*-value	Heterogeneity *P*-value	I^2
PFS	Fixed-Effects Model	3	0.57	0.50	0.64	9.07	<0.001	0.455	0.0%
	Random-Effects Model	3	0.57	0.50	0.64	9.07	<0.001	0.123	0.0%
OS	Fixed-Effects Model	3	0.64	0.55	0.75	5.79	<0.001	0.027	72.4%
	Random-Effects Model	3	0.61	0.44	0.84	2.99	0.003	0.027	72.4%
ORR	Random-Effects Model	3	1.63	1.44	1.84	7.65	<0.001	<0.001	88.5%
	Fixed-Effects Model	3	1.82	1.13	2.91	2.47	0.013	<0.001	88.5%

PFS, progression-free survival; OS, overall survival; ORR, objective response rate; HR, hazard ratio; RR, relative risk; LL, lower limit; UL, upper limit
